# The Role of Bromodomain Testis-Specific Factor, BRDT, in Cancer: A Biomarker and A Possible Therapeutic Target

**DOI:** 10.22074/cellj.2017.5060

**Published:** 2017-05-17

**Authors:** Ekaterina Bourova-Flin, Florent Chuffart, Sophie Rousseaux, Saadi Khochbin

**Affiliations:** CNRS UMR 5309, Inserm, U1209, University of Grenoble Alpes, Institute for Advanced Biosciences, Grenoble, France

**Keywords:** BRD2, BRD3, BRD4-NUT, P-TEFb, iBET

## Abstract

Cancer cells have recently been shown to activate hundreds of normally silent tissue-restricted genes, including a specific subset associated with cancer progression and poor prognosis. Within these genes, a class of testis-specific genes designed as cancer/testis, attracted special attention because of their oncogenic roles as well as their potential use in immunotherapy. Here we focus on one of these genes encoding the testis-specific member of the bromodomain and extra-terminal (BET) family, known as BRDT. Aberrant activation of *BRDT* was first detected in lung cancers. In this study, we report that the frequency of *BRDT’s* aberrant activation in lung cancer varies according to the histological subtypes and in contrast with other cancer/testis genes, it is rarely expressed in other solid tumours. The functional characterization of BRDT in its physiological setting in male germ cells is now painting a clear portrait of its normal activity and also suggests possible underlying oncogenic activities, when the gene is ectopically activated in cancers. Also, these functional studies of BRDT point to specific anti-cancer therapeutic strategies that could be used to “high-jack” BRDT’s action and turn it against cancer cells, which express this gene. Finally, BRDT’s expression could be used as a biomarker for cell sensitivity to BET bromodomain inhibitors, which have become newly available as anti-cancer drugs.

## Introduction

Spermatogenesis is a complex differentiation process involving roughly three phases. First adult stem cells, known as spermatogonia (Fig phase 1), continuously give rise to cells known as spermatocytes, which undergo meiosis (phase 2). Meiosis generates haploid post-meiotic cells (spermatids) that differentiate into spermatozoa (phase 3), the mature and highly specialized male germ cells whose task is to transport the male genome out of the body and, upon fertilization, deliver it to the oocyte. The process of differentiation of the spermatogonia into spermatocytes, then spermatids and eventually mature spermatozoa is controlled by a series of tightly regulated gene expression programs that are activated and repressed in a stage-specific manner (1). Our previous studies have demonstrated the existence of a group of genes that are normally kept silent in all somatic (non-germline) cells and tissues, as well as in all the corresponding developmental stages, including embryonic and stem cells, and shown that most of these genes are male germ cell specific genes (2,3). Indeed, many of the genes expressed in male germ cells have a highly specific pattern of expression, since their activity is exclusively restricted to male germinal cells. We also showed that the repression of these genes in somatic tissues is associated with a specific epigenetic signature on their regulatory regions, which differs from the usual "epigenetic hallmarks" of gene regulatory regions. Also a subset of these genes bears regulatory sequences at their promoter regions that present distinctive and specific features, not shared by housekeeping or other tissue-specific genes (3). 

The reason for the silencing of these genes in somatic tissues is that many of them encode factors that control specific activities that are unique to germ cells and would be harmful to other cell types. For instance, meiosis involves specific and unique events, which include the occurrence of large-scale recombinations as well as a chromosome-wide transcriptional silencing affecting X and Y chromosomes, which are all driven by testis-specific factors. In post-meiotic cells, one of the most dramatic events of chromatin remodelling and genome reorganization takes place. It is characterized by the almost genome-wide removal of histones and their replacement by basic small non-histone proteins, transition proteins (TPs), and finally by protamines (Prms). Here again the products of a subset of testis-specific genes, including a series of histone variants, are exclusively expressed in male germ cells (4). Epigenetic deregulations occur systematically in all cancers and result in changes in gene expression patterns (5). The most studied aspect of these epigenetically driven altered gene expression in cancer is the aberrant silencing of important regulatory genes such as tumour suppressor genes (6). A less studied consequence of these deregulations remains the awakening of normally silent tissue-specific genes (7-9). Following our previous investigations, we observed that cancer cells express hundreds of these tissue-specific genes, and found gene expression signatures associated with highly aggressive tumours in lung cancer, lymphoma and acute lymphoblastic leukemia (ALL) (3,10-12). 

Very little is known on the functional impact of this cancer-associated aberrant "de-silencing" of tissue- specific genes. The activation of testis-specific genes in cancer has been noticed long ago by investigators who were trying to identify cancer specific antigens (8,13). Since then, many male germ cell specific genes have been found aberrantly active in a variety of cancers, and have been classified as "cancer/testis" (C/T) genes. A dedicated site (CT Gene Database: http://www.cta.lncc.br/) now lists C/T genes identified by different groups in various cancers. However, it is worth noting that this list is not exhaustive since many genes not listed on this site are also normally silent in adult non-germline tissues and can be aberrantly activated in a variety of cancers (2,3). The C/T genes could be of particular interest to understand malignant transformation and improve the management of cancer patients. First, their out-of-context activities can initiate unknown oncogenic mechanisms (3,10-12,14,15). The understanding of these mechanisms could also lead to the development of new anti-cancer therapeutic strategies (10,12). Second, the detection of the expression of these normally silent genes in tumours could be used as a specific indicator of the presence of a malignant transformation and hence the products of these genes could constitute good cancer diagnosis biomarkers (2,3,16). Additionally, the involvement of these C/T factors in oncogenic mechanisms can explain the tight association between the expression of some C/T genes and prognosis (9-11). A subset of C/T could also be considered as excellent biomarkers to predict a response to anti- cancer treatments (10,11). Finally, the aberrant activation of these genes reflects the occurrence of upstream epigenetic deregulations. Hence any change in the epigenetic status of this specific category of genes could be considered as indicative of the nature of the epigenetic deregulations that occur in the studied tumours. 

In this review, we will focus our attention on *BRDT*, encoding the testis-specific member of the double bromodomain containing proteins of the BET family. *BRDT* has been shown to be aberrantly activated in a subset of lung tumours (17). Here, we confirm that *BRDT* is indeed activated in lung cancers and it is also activated in other solid tumours, including breast cancer, although less frequently. Our longstanding functional studies of BRDT could help to better understand the oncogenic activities of BRDT, when the gene is aberrantly expressed in cancers, and could also be a basis for developing specific therapeutic approaches targeting tumours that express BRDT. 

### BRDT is a specific remodeler of hyperacetylated chromatin

The first molecular analyses of Brdt showed that its two bromodomains are functional and are specifically capable of binding acetylated histone tails. Also these studies demonstrated that Brdt can induce a dramatic compaction of hyperacetylated chromatin for the first time. This chromatin compaction activity is enhanced after removal of a portion of Brdt C-terminal region (18). Subsequent structural studies of both bromodomains of Brdt revealed outstanding features of its first bromodomain (BD1). Indeed, for its binding, BD1 requires the acetylation of two specific lysines on the H4 N-terminal tail, K5 and K8. The acetylation of either one of these lysines is not sufficient to mediate Brdt histone binding (19). According to the "zip model", histone H4 tail acetylation would spread from K16 to K5, and therefore acetylation of both H4 K5 and K8 can be considered as a signature of the hyperacetylated form of H4, with acetylation of the four acetyl acceptor lysines of H4 (20-22). Interestingly, an independent approach based on the construction of a Brdt-H4 fusion fluorescence resonance energy transfer (FRET) probe, demonstrated that the interaction between Brdt’s BD1 and H4K5acK8ac also occurs *in vivo* (23). 

Very recent investigations of the interactions between BRDT and the nucleosome revealed additional and unexpected properties of BRDT’s BD1. Indeed, these studies showed that BD1 not only binds to H4K5acK8ac but also presents an unexpected DNA-binding activity, which enhances the ability of BRDT to bind to nucleosomes with considerable affinity and specificity. Therefore, both acetylated H4 binding and DNA binding by BD1 are indispensable to observe the acetylated chromatin compaction and genome reorganization by BRDT. BRDT’s second bromodomain (BD2) is not able to bind acetylated nucleosomes (24). This observation is in perfect agreement with our previous observations that Brdt’s BD2 only plays a minor role in the ability of the protein to compact hyperacetylated chromatin (18). These structural and biochemical molecular analyses actually explain many of the properties of Brdt observed in the physiological context of spermatogenesis. 

### BRDT is a driver of meiotic and post-meiotic gene expression, and histone-to-Prm exchange during spermatogenesis 

**Fig.1:**
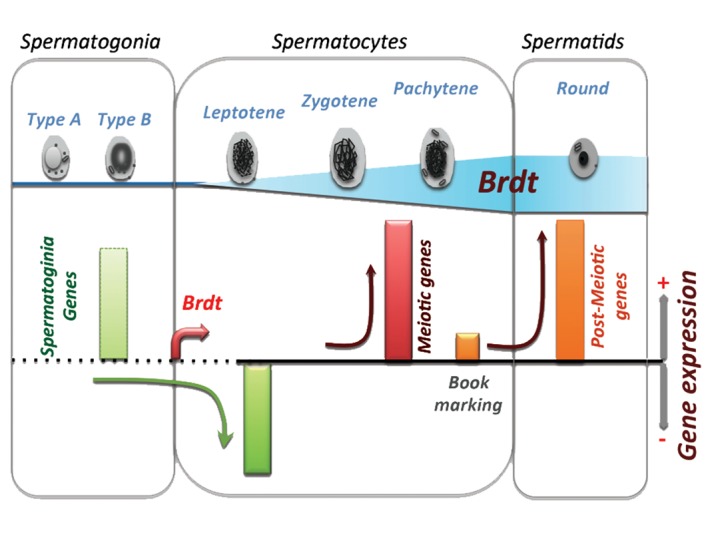
Brdt activation and functions during spermatogenesis. The Brdt gene becomes active when cells commit into meiotic division. In spermatocytes, Brdt directly or indirectly represses a group of genes that were active at the preceding stage in spermatogonia. In spermatocytes, Brdt activates a subset of meiotic specific genes and binds to the transcriptional start sites (TSS) of another group of genes that will become active later, after meiosis. In post-meiotic cells these genes remain fully active until the general shut down of transcription before the genome-wide histone removal. During histone eviction, Brdt’s first bromodomain plays a crucial role in the assembly of transition proteins and the removal of histones.

*BRDT* gene is silent in all somatic tissues (3) as well as in spermatogonia. During spermatogenesis, the gene becomes active at the onset of meiotic cell differentiation, which is highly expressed in spermatocytes and then remains active all through the subsequent stages (Fig.1) (1). The use of different mouse models bearing homozygote inactivation of Brdt (1) or expressing a truncated form of *Brdt* lacking its BD1 (1,25), or even expressing a dominant negative form of Brdt, demonstrated that Brdt drives stage-specific activities. The early activation of a dominant negative form of Brdt at the onset of meiotic differentiation leads to the occurrence of apoptosis and a dramatic loss of cells affecting early spermatocytes. In the total absence of Brdt, an arrest of spermatogenesis is observed at a later stage, in pachytene cells, before the first meiotic division. Interestingly, in mice expressing a mutated form of Brdt lacking its first bromodomain, spermatogenic differentiation occurs normally until the time of histone-to-Prm replacement in post-meiotic cells. Indeed, in these mice, late spermatogenic cells, condensing spermatids and cells at subsequent stages disappear. Our detailed analysis of this mutant showed that in mice expressing Brdt lacking its first bromodomain, histone removal is defective and TPs and Prms, although normally produced, accumulate around the nucleus and are unable to replace histones (1). 

The observation of an earlier and more dramatic effect on spermatogenic differentiation in our dominant negative Brdt mutant mice compared to those totally lacking Brdt (Brdt-KO), suggests that, in absence of Brdt, a compensation occurs up to the pachytene stage, whereas this compensative mechanism is blocked by the dominant negative form of Brdt. Therefore Brdt-specific activities start at the pachytene stage, where no compensation could occur. It is likely that, before this stage, another member of the BET family, probably BRD4 (26), ensures some of Brdt’s functions, and would be inhibited by the dominant negative Brdt mutant. 

The total absence of meiotic defects when spermatogenic cells express a Brdt missing its BD1, shows that all the meiotic and early post-meiotic activities of Brdt are independent of its BD1. 

Our structural studies demonstrated that BD1 is a specialized bromodomain that binds hyperacetylated H4 (19,24). We and others previously showed that histone removal is associated with the occurrence of large-scale histone hyperacetylation during spermatogenesis (27). The structural data on Brdt BD1’s ability to bind hyperacetylated H4 therefore strongly suggest that, in the absence of Brdt’s BD1, the defects should become visible when hyperacetylated histones accumulate. This is precisely what we observed when we analysed hyperacetylation and histone removal in mice expressing Brdt lacking its BD1 (1). 

Our transcriptomic analyses of spermatogenic cells lacking Brdt or expressing Brdt missing its BD1, demonstrated that Brdt is a major driver of meiotic and post-meiotic gene expression programs (1). Many of these genes regulated by Brdt contain H4K5acK8ac at their transcriptional start sites (TSS), suggesting that Brdt recognizes acetylated histones at their TSS and somehow ensures their enhanced expression (28). 

### Functional similarities between BRDT and BRD4

BRD4 is the most studied member of the BET bromodomain-containing factor family. More specifically, the availability of small molecule inhibitors of BET bromodomains attracted great interest on BRD4 and on its role in cancer (29). Early functional studies of BRD4 revealed its association with the Positive Transcription Elongation Factor (P-TEFb) (30) playing a determinant role in the regulation of transcription by the RNA polymerase II (pol II) (31). A detailed analysis of the BRD4-P-TEFb interaction showed that a segment of the C-terminal region of BRD4 is involved in the recruitment of P-TEFb. The same segment is also present in BRDT and transfection experiments demonstrated that it also mediates the recruitment of P-TEFb (32). Following these observations, we showed that endogenous Brdt also interacts with P-TEFb in spermatogenic cells hence explaining the observed transcriptional activities of Brdt (1). 

The *BRD4* gene produces splice variants, which are shorter than full-length BRD4 and lack the P-TEFb-binding domains. A recent investigation demonstrated that the shorter variant of BRD4 can interact with acetylated chromatin and mediate its compaction (33). We reported exactly the same property for a shorter form of Brdt in 2003. A Brdt fragment lacking its C-terminal region acts very efficiently to compact acetylated chromatin both *in vivo* or *in vitro* when purified Brdt was used (18). Therefore, the deletion of Brdt’s C-terminal domain would be a mean to transform a transcriptional regulator into a direct chromatin reorganizer. 

Finally, another similarity between BRDT and BRD4 is that they are both able to remain associated with acetylated chromatin on mitotic chromosomes (18). Indeed, most transcription factors and chromatin binding proteins are released from chromatin during the chromatin compaction associated with mitosis. BRD4 is one of the rare factors that remains bound to chromatin despite the extreme chromatin compaction that occurs during this process. This property of BRD4 underlies one of the most interesting functions of BRD4, which is gene bookmarking and facilitates gene expression after mitosis in cells at the G1 phase of the cell cycle (34). Our ChIP-seq mapping of the Brdt-chromatin interaction demonstrated that Brdt ensures a similar role by bookmarking a subset of genes during meiosis, which will become active in post-meiotic cells (Fig.1). In conclusion, considering the numerous functional similarities between BRD4 and BRDT, one can propose that BRDT is a germ cell-specific BRD4-like factor with some redundant but mostly specific functions. 

### *BRDT* is ectopically activated in solid cancers

**Fig.2:**
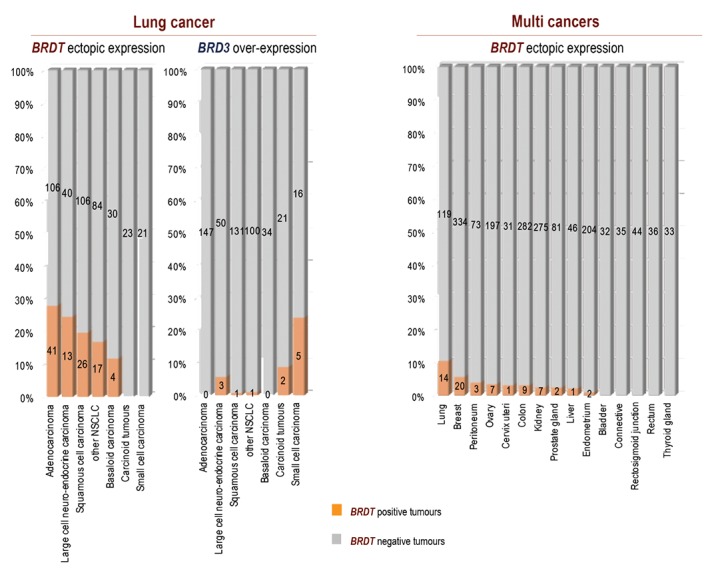
Frequency of BRDT ectopic expression in lung tumours and in various solid tumours. A. For lung cancer, data from three studies were used (GSE30209, GSE8894 and GSE19188, n=511) and B. The data from 1888 solid tumours (GSE21209) were used to detect the ectopic expression of BRDT. Only the subsets of tumours represented by at least 30 samples are shown. In A and B, the bars are labelled with the number of tumours found respectively negative or positive for BRDT expression.

An early work on the ectopic activation of *BRDT* reported the aberrant activation of this gene in 12 out of 47 cases of non-small cell lung cancers (17). However, these authors did not detect any activation of *BRDT* in melanoma, colon, breast, kidney or bladder tumours. The investigation of *BRDT* expression in 511 lung tumours from our own series (3) [GSE30219], as well as from two unrelated published studies (35) [GSE19188] and (36) [GSE8894] allowed us to consider *BRDT’s* activation in specific lung tumour subtypes. Figure 2A shows that 20% of all the analysed lung tumours of this series aberrantly expressed *BRDT* (Fig.2A left panel). The detailed consideration of tumour subtypes in this series revealed large differences in the frequencies of *BRDT’s* activation between different subtypes of lung cancer. Indeed, adenocarcinoma, large cell neuroendocrine tumours and squamous cell carcinoma most frequently activated BRDT (28, 25 and 20% respectively), whereas only 12% of basaloid tumours expressed *BRDT* and none of the carcinoid tumours or the small cell carcinomas of our series expressed this gene (Fig.2A). The somatic members of the same family did not share this pattern of expression (since these are not normally completely silent, we looked for their overexpression in lung cancer). For instance 24% of the small cell carcinoma and 8.7% of the carcinoid tumours of the same series of patients overexpressed the gene encoding BRD3, one of these non testis-specific member of the BET family. In contrast with BRDT, the BRD3 gene was never or very rarely activated in the other histological forms (Fig.2A right panel). In these same patients the overexpression of the *BRD2* or *BRD4* genes was only found exceptionally (one patient on the whole series for each gene). 

We also considered a large series of transcriptomic data corresponding to 1888 tumours belonging to 15 different types of cancer (from GEO publicly available data in GSE2109). We confirmed that lung cancers are the solid tumours ectopically expressing *BRDT* at the highest frequency (11%) (Fig.2B), although this gene was less frequently activated in this series of lung tumours than in the series studied above. This could be due to differences in the proportions of histological subtypes between the two series. Only a small fraction of non-lung solid tumours expressed *BRDT*, with breast tumours expressing *BRDT* at the highest frequency (5.5%). This analysis highlights the propensity of lung tumours compared to other tumour types to aberrantly activate *BRDT*. 

### *BRDT*: a biomarker and a drug target in cancer 

Considering all the properties of Brdt, discovered following molecular and biochemical characterizations, or *in vivo* in the frame of spermatogenic cell differentiation, an important question to address is the functional impact of aberrant *BRDT* activation in an out-of-context situation, in somatic cells, and its contribution to malignant transformation. As mentioned above, BRDT has the same capacity as BRD4 to recruit P-TEFb but its first bromodomain, BD1 shows a strict specificity toward nucleosomes bearing H4K5acK8ac (24). These histone modifications are normally abundant on active gene TSSs (28). The addition of BRDT in this context would increase the intranuclear concentration of BET factors targeting active gene TSSs. Therefore, the ectopically expressed BRDT could add up to the existing BRD4, normally expressed in somatic cells, thereby increasing the number of BET factor-stimulated genes in the cells. However, in spermatogenic cells normally expressing both Brd4 and Brdt, the two factors show different patterns of binding to acetylated chromatin (37). This is consistent with our observation that, despite the expression of Brd4, which temporarily and partially compensates for the absence of Brdt, Brdt-KO spermatogenic cells show altered gene expression and stop at the pachytene stage (1). This observation demonstrates that there is little functional redundancy between Brd4 and Brdt in terms of gene expression regulation. Similarly, it is plausible that BRDT-expressing cancer cells express a different set of genes than those normally regulated by BRD4. 

Short forms of BRD4 and BRDT efficiently induce compaction of acetylated chromatin (18,33). Our data show that even the full-length BRDT has the ability to induce some degree of chromatin compaction (18). Therefore, a possibility would be that, in BRDT-expressing cancer cells, the organisation and dynamics of chromatin may change. This could also be associated with subsequent alterations in gene expression. Interestingly, we also observed that a treatment of BRDT-expressing cells with the histone-deacetylase (HDAC) inhibitor, trichostatin A (TSA), leading to a genome-wide histone hyperacetylation, induces a dramatic chromatin compaction (1,18,24,38). Similarly, it can be foreseen that the treatment of BRDT-expressing cancer cells would induce chromatin compaction at a level that could be fatal to the cells. Accordingly, tumour cells actively expressing BRDT, could be specifically highly sensitive to a treatment including HDAC inhibitors. Finally, since efficient inhibitors of BET bromodomains have been developed and are now in clinical trials in a variety of cancers (39,40), one can also propose that the subset of tumours ectopically expressing BRDT could be good candidates for a treatment with BET bromodomain inhibitors. Indeed, if BRDT activates a set of cancer-specific genes, such inhibitors would counteract the effects of *BRDT* expression. 

## Conclusion

Out-of-context activities of tissue-specific factors directing specialized functions in their physiological settings could initiate a series of unexpected events that would normally never happen in the tissue-of-origin of the corresponding cancer. Within these new activities, some could be the source of or contribute to malignant transformation. Therefore, these ectopic activations could be a source of new biomarkers with high value for cancer diagnosis and prognosis . In addition, the products of these genes could constitute unique anti-cancer targets. In particular, the druggable subset of ectopically activated gene products could be considered as targets of choice. Any specific drug impairing the function of the product of one of these genes should be of high interest in cancer treatment. Indeed, these drugs, while specifically acting on cancers cells, would have little effects on normal somatic cells, which do not express the targeted gene product. *BRDT* could be such a gene whose product could be inactivated by using a BRDT-specific small molecule inhibitor. However, today there is no specific inhibitor for each of the BET members and the available generalist inhibitor molecules could have various adverse effects. BET inhibitors are already used in clinical trials in different cancers but so far the investigators have only considered the targeting of the somatic BET members. *BRDT* expression has never been taken into account to evaluate the responsiveness of the subset of tumours aberrantly expressing this gene. Additionally, the future development of BRDT bromodomain-specific small molecule inhibitors would give the possibility to specifically target the cancer cells without affecting any of the somatic cells expressing the other members of the BET family and hence circumvent the risks associated with the current BET inhibitors. 

Finally, our functional molecular investigations demonstrated that a dramatic chromatin compaction could be induced in somatic BRDT- expressing cells by treating cells with HDAC inhibitors. This property of BRDT could be also exploited to specifically targeting cancer cells expressing high levels of BRDT. Following this idea, the treatment of patients with HDAC inhibitors would induce a collapse of chromatin and the death of cancer cells leaving the normal cells unharmed. HDAC inhibitors are in clinical trials and in certain cases their use has been approved by the FDA. If preclinical data support the idea that *BRDT*-expressing tumours could be sensitive to HDAC inhibitors, then *BRDT* expression could be considered as a good indicator for an anti-cancer therapeutic approach that include HDAC inhibitors. 
